# Effectiveness of a primary care-based group educational intervention in the management of patients with migraine: a randomized controlled trial

**DOI:** 10.1017/S1463423619000720

**Published:** 2019-12-13

**Authors:** Iñaki Aguirrezabal, Maria Soledad Pérez de San Román, Raquel Cobos-Campos, Estibalitz Orruño, Arturo Goicoechea, Rafael Martínez de la Eranueva, Mercedes Arroniz, Elena Uzquiza

**Affiliations:** 1 San Martín Health Centre, Integrated Health Organization Araba (Primary Care), Álava, Spain; 2 Aranbizkarra II Health Centre, Integrated Health Organization Araba (Primary Care), Álava, Spain; 3 Methodology and Statistics Unit, Bioaraba Health Research Institute, Araba University Hospital, Álava, Spain; 4 Araba University Hospital (Santiago Headquarters), Integrated Health Organization Araba, Álava, Spain; 5 Multidisciplinary Teaching Unit of Araba, Integrated Health Organization Araba, Álava, Spain; 6 Labastida Health Centre, Integrated Health Organization Araba (Primary Care), Álava, Spain

**Keywords:** educational models, chronic migraine, migraine disorders, migraine treatment, neuroscience, primary care

## Abstract

**Aim:**

The aim of this study was to assess the effectiveness of a primary care-based group educational intervention about concepts of pain neuroscience for the management of migraine compared to the routine medical care delivered to patients with this condition.

**Background:**

The way pain is understood has been radically changed in recent decades, thanks to developments in the field of neuroscience. Thus, migraine may develop as a result of an exaggerated perception of threat that activates the pain neuromatrix, which might be modifiable, from a learning perspective, by adjusting the beliefs and behaviours that favour the onset of an attack.

**Methods:**

A randomised controlled trial was carried out in five primary care health centres of Vitoria-Gasteiz (Basque Country, Spain). The follow-up period was 12 months. The main outcome measure was the reduction in days lost due to migraine-related disability according to the Migraine Disability Assessment Test (MIDAS) score. Secondary outcome measures included the intensity and frequency of the pain and the number of analgesic drugs taken in the previous three months. A positive response to treatment was considered when the MIDAS score decreased by at least 50% from baseline.

**Findings:**

Days lost due to migraine-related disability decreased by at least 50% in 68.9% (*n* = 37) of patients in the intervention group and 34.6% of patients in the control group (*n* = 18) (*P* < 0.001). The intensity of the headache [odds ratio (OR) 9.116; *P* = 0.005] and the medication intake (OR 13.267; *P* < 0.001) were also significantly reduced with the intervention.

**Conclusions:**

The provision of suitable information through a group educational intervention delivered in primary care appears to be effective in preventing migraine attacks. Moreover, the intervention could offer a new cost-effective management alternative that seems to reduce the need for pharmacological treatment in patients with migraine.

## Background

Migraine is the sixth leading cause of days lived with disability worldwide (GBDS, 2015). The level of disability due to migraine has increased in recent years, accounting for 1.3% of all years of life lost to disability globally (Liaño-Martínez and Liaño-Riera, 2003; Global Burden of Disease Study 2013 Collaborators, 2015). Moreover, the condition has a negative impact on the quality of life of sufferers (Dahlöf and Dimenäs, 1995). In the USA, the productivity at work or school of individuals with migraine is reduced by at least 50%, given that the prevalence of migraine is higher among people in the most productive years (i.e., from the end of adolescence to the fourth decade of life) (Steiner *et al*., 2003). Migraine is one of the most costly neurological disorders. In 2004, the total costs associated with migraine rose to 27 000 million Euros in Europe alone (Stovner and Andrée, 2008) with an estimated 25 million days of work or school lost yearly due to migraine in the UK and some 190 million in Europe overall (Steiner *et al*., 2003).

The number of medical visits due to migraine in the USA increased from 2.3 to 5 million between 1990 and 1998 and is still rising (Gibbs *et al*., 2003). Nevertheless, despite the numerous pharmacological treatment and prevention options available for migraine attacks, only 40% of patients with migraine are highly satisfied with their current treatment (The National Survey, Migraine in America, 2016). Regarding the pharmacological treatment, it is important to note that 37% patients that had used migraine medication in the past experienced adverse effects (sleepiness, tiredness and difficulty in thinking clearly) (Gallagher and Kunkel, 2003).

The most widely accepted hypothesis for the origin of migraine attacks is based on a state of neuronal hyperexcitability that leads to a cortical spreading depression and the consequent sensitisation of the trigeminovascular system, a necessary requirement for the onset of pain (Zhao and Dan Levy, 2016). Despite scientific advances in our understanding of the molecular processes underlying migraine, outcomes in terms of prevention and control of attacks are not fully satisfactory with current strategies.

In recent decades, there has been a radical change in the way pain is understood. It is currently considered that pain does not originate in the peripheral nociceptors, but rather in a network of brain regions called the pain neuromatrix (Gifford and Butler, 1997; Gifford, 1998; Gifford and Muncey, 1999), whose activation is necessary and sufficient to generate the perception of pain. Such activation can be caused by the arrival of a nociceptive signal or by alert states due to an implicit perception of threat to the body; even though the threat may not be real and thus, it could be modified by adjusting beliefs and behaviours that favour the onset of an attack. Moreover, the fear of pain is a factor closely related to the severity of the headache (R^2^ = 6.1%; *P* < 0.01) and to the disability related to pain (R^2^ = 4.5%; *P* < 0.01) (Black *et al*., 2015). The fear-avoidance model of chronic pain describes how individuals experiencing acute pain may become trapped into a vicious circle of chronic disability and suffering (Lethem *et al*.,^1983^; Vlaeyen and Linton, 2002; Crombez *et al*., 2012; Hasenbring *et al*., 2012).

A pain neuroscience-based educational intervention has shown effectiveness in patients with chronic pain due to fibromyalgia (Van Oosterwijck *et al*., 2013) and lower back radiculopathy (Louw *et al*., 2014), with consequent reductions in pain and in disability. Nonetheless, pain neuroscience education strategies have not been tested in patients with migraine. The scientific literature only gathers evidence about a psychological intervention (which includes relaxation training and cognitive behavioural therapy) in patients with this condition, which appears to be effective for the management of migraine (Sullivan *et al*., 2016). However, the evidence base is still lacking in quality. A recently published update on behavioural treatments for migraine concluded that these types of treatments seem to be as effective as the pharmacological treatment for the prophylaxis of migraine and the observed effects are even more pronounced when the pharmacological and behavioural treatment are applied in combination (Kropp *et al*., 2017).

The aim of the study was to assess the effectiveness of a primary care-based group educational intervention in which patients were trained in the current concepts of pain neuroscience applied to migraine, for the management of the condition, compared to routine medical care.

## Methods

We conducted a parallel randomised controlled trial on 116 patients from five primary health centres located in one of the provinces (Álava) of the Basque Public Health Service (San Martín, Sansomendi, Lakuarriaga, Gazalbide and Zabalgana) diagnosed with migraine (ICD-9-CM Diagnosis Code 346) who had at least one migraine attack per month despite treatment. Patients with mental illness, cognitive impairment or those with difficulties to understand the Spanish language were excluded, because such conditions might hinder completion of the follow-up. Patients that could not attend all sessions of the intervention or had received training as part of the previous pilot study were also excluded.

Patients were recruited between August 2013 and May 2015. The study was approved by the Clinical Research Ethics Committee of the Araba University Hospital on 21 September 2012. All participants gave written informed consent prior to inclusion in the study. The sample size of the study was calculated, and we estimated that a sample of 106 patients was required to detect a difference of at least 25% (19% versus 44%) in the rate of patients with 50% improvement of the Migraine Disability Assessment Test (MIDAS) score between comparison groups (Mérelle *et al*., 2007). Additionally, the sample size was increased by 10% to cover for losses that might occur during the follow-up.

Potential candidates for the study were identified from data entered in the electronic health record system from the Basque Health Service (OSABIDE-PC), selecting patients with a diagnosis of migraine (ICD-9-CM Diagnosis Code 346). Primary care doctors phoned patients to tell them about the study inviting them to participate. Additionally, patients were told that they could continue with the usual pharmacological treatment (i.e., analgesic drugs during attacks or preventive drugs).

After inclusion in the study, patients were randomly allocated (simple randomisation) to the intervention or control groups using a computer-generated randomisation sequence obtained by a third party (independent of the research team) and concealed from the research team until patients were allocated to the study groups. The allocation ratio was 1:1.

### Description of the intervention

The intervention was administered by health professionals, including two family doctors and one neurologist with extensive training and experience in the proposed model and was carried out in the San Martín Health Centre between November 2013 and June 2015. Patients were divided into groups of 10–12 participants to attend five sessions, four given once a week for four weeks and the fifth, 1 month after the fourth session. Each session lasted for 1 h and 45 min.

In all sessions, neuroscience-based information on the neurophysiology of pain and migraine were provided by means of audio–visual support. The following concepts were covered: pain does not equate to injury, perception, pain is generated in the brain, nociception, necrosis, apoptosis and inflammation; congenital and acquired components of defence systems, analogy with the immune system, memory and learning in pain, genetics and the environment; mirror neurons and learning by imitation; cultural learning and learning with experts; reward systems; fear, brain plasticity, placebo and nocebo effects and the efference copy system. In each session, a patient who had participated in a previous session was involved to share his/her experiences and thereby reinforce the message of the intervention.

The following written support material was provided to endorse the information given in the session: two books, “Migraña: una pesadilla cerebral” (“Migraine: a brain nightmare”) (Goicoechea, 2009) and “Explain pain” (Butler and Moseley, 2013) and the blog “know pain, no pain” (Goicoechea, 2017). The intervention was based on other interventions described in clinical trials for other pain conditions (Moseley *et al*., 2004; Louw *et al.*, 2011; Louw *et al.*, 2014).

Patients in the control group received the usual clinical care consisting of periodical primary care appointments. The only difference between both groups was the series of educational sessions provided to the intervention group patients.

### Follow-up

After recruitment, patients were interviewed by research team members in charge of the assessments. During the interviews, the following data were collected: demographic characteristics, beliefs regarding migraine, coping strategies for migraine attacks, the MIDAS questionnaire (Stewart *et al.*, 2001), medication taken, time of work, emergency department attendances and limitation of daily activities due to migraine, during the previous three months.

Further assessments were carried out over the telephone after 3, 6 and 12 months of follow-up. The individuals carrying out the assessment were blinded to group allocation.

### Assessment of the response

At 12 months after the start of the study, we evaluated the primary outcome measure, namely, days lost due to migraine-related disability measured using the MIDAS questionnaire, that is, the sum of responses to five specific questions on the level of disability (days missed or with reduced productivity at work/school, at home and in leisure activities) (Stewart *et al.*, 2001). A positive response to treatment was considered when the MIDAS score decreased by at least 50% from baseline. The Spanish version of the MIDAS questionnaire was used in the study (Fernández-Concepción and Canuet-Delis, 2003).

The secondary outcome measures were the intensity and frequency of the pain, measured using the two additional questions of the MIDAS questionnaire together with the number of analgesic drugs taken in the previous three months. Similarly, a decrease of at least 50% in these parameters compared to baseline was considered to indicate a positive response to treatment. In addition, we assessed the degree to which activities of daily life were limited by migraine, using an *ad hoc* scale with six possible answers: not at all, very little, little, quite a lot, a lot and totally.

In parallel, for all patients we collected data on costs associated with the migraine-related pharmacological treatment, which included both preventive medication and medication for the treatment of migraine attacks. All variables were assessed after 12 months of follow-up.

### Statistical analysis

The main outcome measure (≥50% decrease in the MIDAS score as compared to baseline measurements) was assessed with logistic regression. We constructed a crude model and a model adjusted for potential confounding variables, identified through bivariate analysis of each independent variable with the dependent one. The results were expressed as odds ratios (ORs) with the corresponding 95% confidence intervals (95% CI). We only included in the adjusted model those variables that showed a statistically significant relationship with the dependent variable (≥50% decrease in the MIDAS score). The same model was used to analyse the secondary variables (≥50% decrease in intensity and frequency of pain, as well as in the number of analgesic drugs taken in the previous three months). The goodness of fit of each model was examined using the Hosmer–Lemeshow test, the fit was considered good for values of *P* > 0.05.

The limitation of daily activities due to migraine was assessed using the Chi-square test. For this purpose, we recoded the data into a dichotomous variable, grouping response options into not at all/little/very little, on the one hand, and quite a lot/a lot/totally, on the other hand.

We also carried out an economic analysis, calculating the mean medication expenditure per patient in both groups, and the incremental cost-effectiveness ratio (ICER) (difference in costs/difference in effectiveness) defined as the additional cost per unit of additional benefit associated with the intervention. The unit of effectiveness used for the ICER calculation was the primary outcome measure (≥50% decrease in the MIDAS score from baseline). The ICER was calculated for (1) the total cost of migraine-related medication (i.e., both, for prevention and for management of migraine attacks) and (2) specific cost of medication for the management of migraine attacks. All variables were assessed at 12 months of follow-up.

## Results

**Figure 1.**
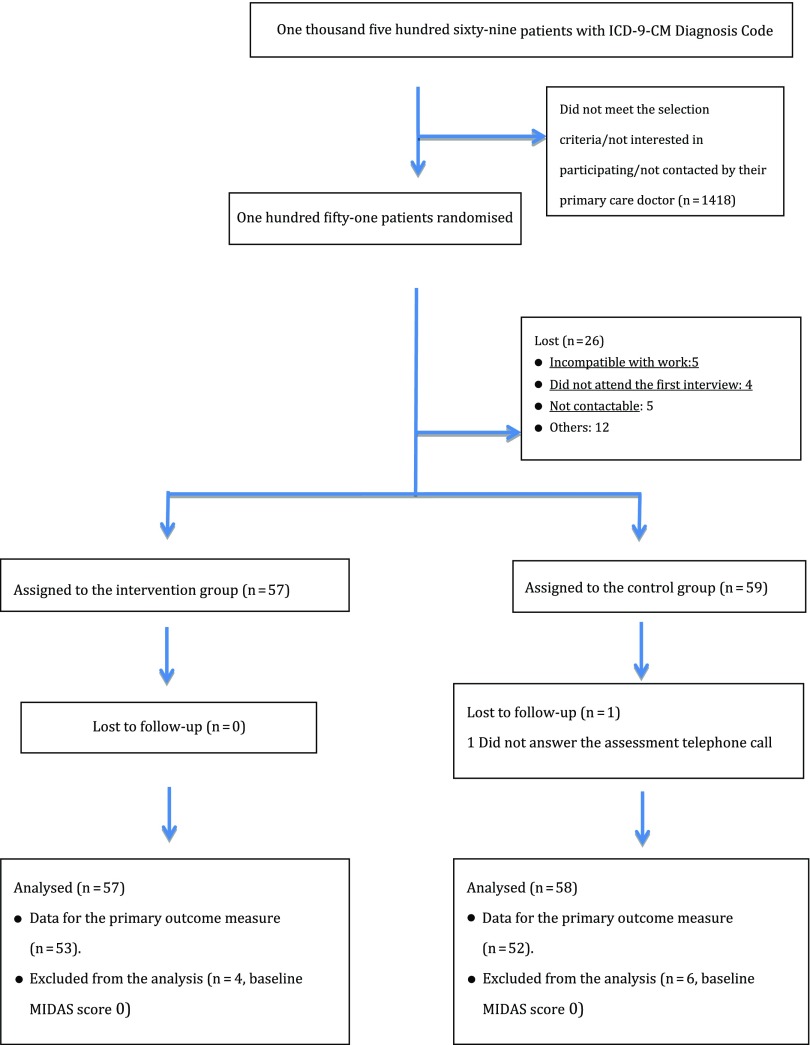
Flow of patients through the study.

In order to assess the effectiveness of an educational group intervention for the management of migraine, we studied 116 patients between November 2013 and June 2015, of whom 115 completed the 12 month follow-up (Figure 1). One patient was lost, due to inability to contact him. Out of the 115 patients who finally participated in the study, only 105 were assessed for the main outcome variable (≥50% decrease in the MIDAS score from baseline), because the other 10 patients obtained a score of zero on the MIDAS questionnaire at the start of the study (Figure 1). Nevertheless, all 115 patients were included in the analysis of the other secondary outcome measures.

Out of the 116 patients included in the study, 57 (49.1%) were assigned to the intervention group and 59 (50.9%) to the control group. Overall, 95 were women (81.9%) and 95 (81.9%) were married or living as a couple. We did not find significant differences in the baseline characteristics between the groups (*P* > 0.005) (Table 1), except for preventive medication intake before the start of the study (nine patients (15.3%) in the control group compared to two patients (3.5%) in the intervention group, *P* = 0.031).

**Table 1.** Baseline clinical characteristics of the study participants

	Intervention group (*n* = 57) N (%)	Control group (*n* = 59) N (%)
Age (years)		
21–30	5 (8.8)	10 (16.9)
31–40	12 (21.1)	16 (27.1)
41–50	18 (31.6)	15 (25.4)
51–60	22 (38.6)	18 (30.5)
Sex, female/male (*n* = 116)	44 (77.2)/13 (22.8)	51 (86.4)/8 (13.6)
Current employees	43 (75.4)	38 (64.4)
Married or living as a couple	44 (77.2)	51 (86.4)
Completed secondary education/university	46 (80.7)	44 (74.6)
Age of migraine onset <20 years of age	29 (50.9)	39 (66.1)
Migraine with aura	20 (35.1)	25 (42.4)
Family history of migraine	40 (70.2)	38 (64.4)
Had been seen by a neurologist (at least once)	29 (50.9)	37 (62.7)
Unknown cause of the migraine	35 (61.4)	32 (54.2)
Took early analgesic treatment	44 (77.2)	40 (67.8)
Daily activities limited during attacks	24 (42.1)	32 (54.2)
Preventive medication	2 (3.5)	9 (15.3)

### *Decrease of* ≥*50% in the MIDAS score (primary outcome measure)*

The MIDAS scores of the five questions related to the level of disability decreased by at least 50% in 68.9% (*n* = 37) of patients in the intervention group and 34.6% of those in the control group (*n* = 18), the difference being statistically significant (*P* < 0.001). In multivariate analysis, the difference between the groups remained significant (OR 4.225; 95% CI 1.826–9.777, *P* = 0.001). The ratio of response to no response to treatment was 4.255-fold higher in the intervention group than among patients in the control group. Interestingly, the intervention was more effective in patients who lived alone (Table 2).

**Table 2.** Effectiveness of the group educational intervention in reducing the MIDAS score by ≥50%

2.1 Univariate analysis			
	≥50% reducing the MIDAS^b^ score	<50% reducing the MIDAS^b^ score	Sig.^c^
Intervention group	69.8%	30.2%	= 0.000
Control group	34.6%	65.4%	

2.2 Multivariate analysis					
	B	Sig.^c^	OR^a^	95% CI for OR	
				Lower	Upper
Intervention	1.441	0.001	4.225	1.826	9.777
Separated/Single/Widow(er)	1.243	0.034	3.466	1.098	10.934
Constant	−0.846	0.007	0.429	–	–

a Odds ratio.

b Migraine Disability Assessment Test.

c Statistical significance.

### Secondary outcome measures

Decreases by at least 50% in the duration (in days) and intensity of headache and in medication intake were assessed and significant differences were observed in all cases in favour of the intervention group (*P* < 0.005, Tables 3–5). The Hosmer–Lemeshow test indicated a good fit for all models (*P* > 0.05 in all cases).

**Table 3.** Effectiveness of the group educational intervention in reducing the duration of the headache (in days) by ≥50%

3.1 Univariate analysis			
	≥50% reducing the headache duration	<50% reducing the headache duration	Sig.^b^
Intervention group	71.9%	28.1%	= 0.000
Control group	22.4%	77.6%	

3.2 Multivariate analysis					
	B	Sig.^b^	OR^a^	95% CI for OR	
				Lower	Upper
Intervention	2.841	0.000	17.135	5.656	51.906
Headache in the previous three months	0.066	0.001	1.068	1.029	1.108
Separated/Single/Widow(er)	1.431	0.029	4.182	1.154	15.156
Constant	−3.117	0.000	0.044	–	–

a Odds ratio.

b Statistical significance.

**Table 4.** Effectiveness of a group educational intervention in reducing the headache intensity by ≥50%

4.1 Univariate analysis			
	≥50% reducing the HEADACHE intensity	<50% reducing the HEADACHE intensity	Sig.^b^
Intervention group	24.6%	75.4%	= 0.001
Control group	3.4%	96.6%	

4.2 Multivariate analysis					
	B	Sig.^b^	OR^a^	95% CI for OR	
				Lower	Upper
Intervention	2.210	0.005	9.116	1.966	42.268
Constant	−3.332	0.000	0.036		

a Odds ratio.

b Statistical significance.

**Table 5.** Effectiveness of the group educational intervention in reducing the medication intake by ≥50%

5.1 Univariate analysis			
	≥50% reducing the medication intake	<50% reducing the medication intake	Sig.^b^
Intervention group	73.7%	26.3%	= 0.000
Control group	22.8%	77.2%	

5.2 Multivariate analysis					
	B	Sig.^b^	OR^a^	95% CI for OR	
				Lower	Upper
Intervention	2.585	0.000	13.267	5.115	34.415
Absolute number (baseline)	0.017	0.011	1.017	1.004	1.031
Constant	−1.931	0.000	0.145	–	–

a Odds ratio.

b Statistical significance.

Migraine had a greater limiting impact on the life of patients in the control group than those in the intervention group, with a significant difference between the groups. Specifically, 32 control group patients (55.2%) and just six intervention group patients (10.5%) reported that migraine limited their daily activities quite a lot, a lot or totally at 12 months of follow-up (*P* < 0.001).

### Economic analysis

The mean expenditure per patient on medication to treat migraine attacks was €45.53 in the control group and €12.33 in the intervention group. Including the cost of preventive medication, the figures were €53.91 and €12.33 for the control and intervention groups, respectively (Table 6).

**Table 6.** Economic analysis

	Costs	
	Control (*n* = 58)	Intervention (*n* = 57)
Cost of medication for the treatment of migraine attacks (€)	2641	703
Total cost (both, for prevention and treatment of acute attacks) (€)	3127	703

### Cost-effectiveness analysis

For the cost-effectiveness analysis, we only included 105 patients, namely, those who had a baseline MIDAS score greater than zero. Taking into consideration the cost of drugs for the prevention and treatment of acute migraine attacks, the ICER was −€92. This means that with the educational group intervention €92 can be saved for each additional patient whose MIDAS score decreases by at least by 50% (unit of additional benefit) (Table 7).

**Table 7.** Incremental cost-effectiveness ratio

		Effectiveness	
		Control (*n* = 52)	Intervention (*n* = 53)
50% decrease in the MIDAS score	No	34	16
	Yes	18	37
Cost of medication to treat migraine attacks (€)		1956	702
Total cost of medication (both, for prevention and treatment of acute attacks) (€)		2441	702

## Discussion

Advances in the management of migraine are not sufficient to improve the quality of life of sufferers, and new therapeutic strategies are urgently needed to achieve better outcomes. The purpose of this investigation was to explore such therapeutic alternatives for people with migraine. Namely, the present study aimed at assessing the efficacy of an educational group intervention on pain neuroscience for the treatment and management of migraine. The intervention was more effective than usual care at 12 months of follow-up in achieving a 50% decrease or greater in the MIDAS score (OR 4.225; *P* = 0.001), intensity of pain (OR 9.116; *P* = 0.005) and medication intake (OR 13.267; *P* < 0.001). In the light of the results derived from the present study, the group educational intervention tested could offer a new cost-effective management alternative that appears to reduce the need for pharmacological treatment in patients with migraine.

The findings of this study are in agreement with those obtained by other research groups. Rothrock *et al.* (2006) carried out a randomised controlled trial to assess the effectiveness of an educational intervention in patients diagnosed with migraine compared to standard care. Patients from the intervention group received written information about the biogenesis and treatment of migraine, together with three peer-to-peer sessions guided by patients who suffered from migraine and had previously been taught about the biogenesis, prevention and treatment of the condition. The researchers observed a significant difference in the change of the MIDAS scores from baseline. Specifically, scores decreased by 24 points after 6 months of follow-up in the intervention group as compared to 14 points in patients from the control group. The pattern of decrease was similar to that found in the present study (11.2 compared to 0.4). The difference in magnitude could be attributed to the fact that we assessed the outcome measure after 12 months of follow-up.

As mentioned above, there has been some research on educational interventions for the management of migraine (Rothrock *et al*., 2006). Nevertheless, no studies were identified on pain neuroscience-based educational interventions. These type of educational interventions on pain neuroscience have, however, been previously investigated for other conditions such as, fibromyalgia and lumbar radiculopathy and were found to be effective (Van Oosterwijck *et al*.,^2013^; Louw *et al*., 2016). Current evidence supports the use of education in neuroscience for the reduction of chronic musculoskeletal pain, through a better knowledge about pain by the patient, which, in turn, improves movement and minimises the use of healthcare services (Louw *et al*., 2016).

For the intervention tested in the present study, we have applied a conceptual framework developed by other authors for chronic low back pain (Gifford and Butler, 1997; Gifford,^1998^; Matchar *et al*., 2008; Mo’tamedi *et al.*, 2012; Sullivan *et al*., 2016) and adapted for migraine by Goicoechea, who proposed that migraine should be regarded as an abnormal perception of threat that triggers the body’s defence system. Thus, the educational intervention described on this paper for chronic pain due to migraine can be regarded as innovative. Briefly, the underlying concept is that if migraine is considered an abnormality in the brain’s perception, with a considerable component of cultural learning, rather than an inevitable consequence of a genetically hypersensitive brain, we can seek to modify that erroneous perception through education. We believe that providing suitable information, the existing knowledge, beliefs and behaviours that favour migraine attacks could be altered, while developing other patterns that are less disabling.

Unlike the studies on pain neuroscience group educational interventions for fibromyalgia and low back pain (Van Oosterwijck *et al*.,^2013^; Louw *et al*., 2016), the present investigation was conducted in primary care. This level of care might be the most appropriate to deliver these types of interventions. It is important to highlight that group educational interventions are common in the primary care setting (e.g., for patients with diabetes, chronic obstructive pulmonary disease, smokers, etc.) and, generally, primary care professionals are experienced in these type of interventions. Furthermore, we think that patients might perceive primary care professionals to be more accessible than other hospital-based specialists.

### Strengths and limitations of the study

The study was designed to maximize the internal and external validity. On the one hand, patients were randomly assigned to one of the two study groups, through a computer-generated randomisation sequence, concealed from researchers until group allocation. Prognostic and potential confounding factors were thus distributed in a balanced manner between the study groups. On the other hand, the follow-up was carried out by blinded researchers, who did not participate in the recruitment phase and were unaware of group allocation. As a consequence, the internal validity of the study was reinforced. In this regard, other authors have pointed out that a lack of blinding may lead to systematic differences in the medical care provided to patients. For studies in which researchers cannot be blinded, Sutton *et al.* (2013) proposed that at least some follow-up visits should be recorded and subsequently compared.

The external validity was also strengthened by the fact that the research team was composed of highly qualified health professionals, who were responsible for the follow-up and management of patients with migraine in their everyday clinical practice. Moreover, the sample of patients in the study was highly representative of the target population, which allows generalisation of the findings.

Very few investigations evaluating preventive treatments for migraine have performed assessments after 12 months from the beginning of the study. Indeed, most studies have followed up patients for six months at most. We believe, however, that demonstrating the effectiveness of a chronic disease such as migraine requires at least one year of follow-up to properly monitor the response to treatment.

The main limitation of the present study is that due to the nature of the intervention, patients could not be blinded. Such lack of blinding could have an impact on the results, since the answers to the MIDAS questionnaire are subjective. However, we would also like to point out that this is a common limitation of clinical trials assessing educational interventions.

Another limitation could be the fact that the effect of group dynamics on the outcomes was not controlled. It was, therefore, not possible to determine whether the treatment outcomes were a consequence of the group dynamics or of the educational material about neuroscience delivered during the sessions, since there was no comparable group dynamic opportunity in the control group. In similar studies, in which group educational or psychological interventions were assessed, no specific intervention was delivered to the participants in the control group apart from the usual medical care (Gifford and Muncey, 1999; Matchar *et al*., 2008; Mo’tamedi *et al*., 2012). Further, regardless of the effectiveness of the individual underlying components of the intervention, we considered that it was important to test the global effectiveness of the group educational intervention as compared to usual care.

## Conclusion

In the light of the results derived from the present investigation, a group-based educational intervention could be an effective strategy for the management of migraine, since it substantially reduces the number of days lost due to the condition, diminishes the intensity of pain and decreases the medication intake. We believe that primary care could be an appropriate setting for this type of group educational intervention.
